# The Needs of the Journal

**Published:** 1915-10

**Authors:** 


**A Monthly Review of Medicine and Surgery**

EDITOR AND PUBLISHER, DR. A. L. BENEDICT, 228 Summer St., corner of Elmwood Ave., Buffalo, (Address for all communications. Please make personal and telephone calls before 1 P. M.)

Third, in age, on the western continent. Independent, but supports professional organization. News limited mainly to Western N. Y. and Penn.

Subscription $2.00 a year; $3.00 for two years in advance. Changes and errors in address or failure or duplication of delivery, should be reported immediately.

Yearly Volume 71 OCTOBER, 1915 No. 3

### The Needs of The Journal.

A comparison of the year recently past with previous ones, shows a healthy, gradual gain in financial support. Some minor economies have also been accomplished, as practical experience of various kinds has been acquired. On the other hand, it has not been considered desirable to regard the Journal as a money-making business, but rather as an institution serving a professional end and, on this account, greater liberality has been considered safe in extending its usefulness in various ways. These statements should be qualified to refer to conditions presupposing a prompt receipt of subscriptions. It is not desirable on any account to lower expenses and thus lower the quality of the Journal, so as to render it independent of due support.

So far as Buffalo and surrounding towns are concerned—with due regard for subscription sharing—there is very little to be desired or expected in the way of increased circulation. Fortunately, the medical profession is not growing to any appreciable extent in numbers. Without any special effort, the scattering, distant circulation is gradually increasing. For the near future, it seems advisable to conduct the Journal mainly as a local organ—not for Buffalo, but for Western New York, a district that corresponds in general and professional population to about two average states or about one-twentieth of the whole U. S. It should be understood that it is not economically possible to conduct a professional journal, on a self-supporting basis, at a reasonable subscription rate, for any such number of persons as are contained in any one city, or state, or other area having a population much smaller than that mentioned. It may be frankly admitted that the Journal does need greater support from the southern and eastern portions of its territory and we would be pleased to have our readers bear this in mind as they meet members of the profession. We may also suggest that unless the journal is saved for binding, an occasional copy might be mailed to some professional friend who is not a subscriber.

As it is obviously impracticable for a journal of this scope to employ reporters and correspondents, we are dependent for accuracy in news items of various kinds, upon the co-operation of our readers and more particularly for distant points and those in which we have no associate editor. This statement applies especially to personal and obituary notices.

While the Journal is not directly in need of funds, there are various fields of usefulness which might be cultivated if special endowments were available. For example, reports of institutions and societies ,of prohibitive length and expense in printing considered simply as matter for supplying its readers in general, could be given a wider range and could be preserved if it were of sufficient interest to the members of these institutions and societies to pay for the direct expense. Likewise, professions more or less closely affiliated with the medical profession or organizations carrying on educational campaigns not too far remote from medicine, might find it advantageous to arrange for the regular publication. of a section in the Journal.

The Journal has just about enough original matter contributed. Without admitting that this matter has not been of a high grade, it is true as of every human enterprise, that improvement is always possible. We feel that, for selfish motives as well as altruistic, the physician's best and most practical work should be published where it will most easily reach his immediate professional colleagues. Illustrations always enhance the interest if not the actual value of original articles.

A large amount of valuable matter in domestic and foreign exchanges is wasted, simply for the reason that no one man and no small number of men can read an average of ten journals a day, especially when we consider that some are in foreign languages. The editor can easily, in the course of reading necessary for his own practical use, prepare enough abstracts to fill whatever space is available, but it would be far better to have the broader judgment of a number of reviewers, interested in a number of special lines. For obvious reasons, most additional review work must be done by members of the local profession.

It is an open secret of journalism that while subscriptions maintain the circulation, most of the nutrition must be derived from advertising. With no special reference to this journal nor even to professional journals in general, we ask you to bear in mind that circulars are the bane of all legitimate publications and that the reason that you can buy a cent's worth (wholesale) of paper in the form of a penny newspaper and that the average subscription rate of all sorts of periodic literature is about half the actual cost, is due to your attention to regular advertising. If we carry advertising that is not proper, let us know. If there is any advertising in your vicinity that is suitable to our columns and that we do not carry, please inform us and also speak to the potential advertiser about it. If we can increase our average advertising to 50 pages, inclusive of indexes, and "readers" proportionate to actual advertising, we can reduce the subscription to $1.50.

In speaking thus candidly about the journal, we are justified by the fact that it was assumed by the present editor in the spirit of its inception and subsequent maintenance, namely that it is essentially a matter of mutual, co-operative professional labor. It is a paradox that such a spirit is pre-eminently necessary for a nominally independent journal.

